# Gamma-Irradiation-Induced Electrical Characteristic Variations in MoS_2_ Field-Effect Transistors with Buried Local Back-Gate Structure

**DOI:** 10.3390/nano14161324

**Published:** 2024-08-07

**Authors:** Su Jin Kim, Seungkwon Hwang, Jung-Dae Kwon, Jongwon Yoon, Jeong Min Park, Yongsu Lee, Yonghun Kim, Chang Goo Kang

**Affiliations:** 1 Korea Atomic Energy Research Institute, 29 Geumgu-gil, Jeongeup 56212, Republic of Korea; sujink@kaeri.re.kr (S.J.K.);; 2 Energy and Environment Materials Research Division, Korea Institute of Materials Science (KIMS), 797 Changwondaero, Sungsan-gu, Changwon 51508, Republic of Korea; hhs0505@kims.re.kr (S.H.);

**Keywords:** MoS_2_, two-dimensional materials, buried local back-gate, gamma irradiation, field-effect transistor

## Abstract

The impact of radiation on MoS_2_-based devices is an important factor in the utilization of two-dimensional semiconductor-based technology in radiation-sensitive environments. In this study, the effects of gamma irradiation on the electrical variations in MoS_2_ field-effect transistors with buried local back-gate structures were investigated, and their related effects on Al_2_O_3_ gate dielectrics and MoS_2_/Al_2_O_3_ interfaces were also analyzed. The transfer and output characteristics were analyzed before and after irradiation. The current levels decreased by 15.7% under an exposure of 3 kGy. Additionally, positive shifts in the threshold voltages of 0.50, 0.99, and 1.15 V were observed under irradiations of 1, 2, and 3 kGy, respectively, compared to the non-irradiated devices. This behavior is attributable to the comprehensive effects of hole accumulation in the Al_2_O_3_ dielectric interface near the MoS_2_ side and the formation of electron trapping sites at the interface, which increased the electron tunneling at the MoS_2_ channel/dielectric interface.

## 1. Introduction

In recent years, two-dimensional (2D) materials, including graphene, hexagonal boron nitride, and transition metal dichalcogenides (TMDs), have been proven to possess excellent properties, demonstrating their suitability for various applications, such as electronics, optoelectronics, sensors, energy storage, catalysis, and biology [1,2,3,4,5]. Among these 2D materials, MoS_2_ has been extensively studied because of its superior properties, including its ultrathin body, high carrier mobility, mitigation of short-channel effects, and tunable bandgap from 1.3 eV for bulk MoS_2_ to 1.8 eV for a monolayer [6,7,8,9,10,11,12,13,14]. These unique properties of MoS_2_ materials qualify them as promising candidates for channel materials in high-performance, downscaled, and low-power electronic devices and specialized radiation environments, such as those relevant to space, defense, and nuclear applications.

For radiation applications, ionizing radiation induced by the interaction between gamma photons and semiconductors can produce electron–hole pairs and diverse defects in the interaction materials, which can affect their structural and electrical properties. Radiation damage degrades the quality of the channel material, which in turn degrades the device performance. Conversely, atomic MoS_2_ with a layer thickness of approximately 0.65 nm offers excellent properties suitable for application in radiation-resistant devices because of its small volume that interacts with radiation [6]. Therefore, an in-depth understanding and analysis of radiation-tolerant technologies and the radiation response of TMD materials (e.g., MoS_2_) are critical steps in implementing radiation-related electronic devices for practical applications.

Although research on the effects of gamma radiation at the MoS_2_ transistor level is still in its initial stages with several technical challenges still to be addressed, experiments aimed at identifying the effects of gamma radiation on MoS_2_-based transistors and films have been conducted. For example, Isherwood et al. reported that high-dose gamma irradiation causes radiation damage, such as the radiolytic oxidation, doping, and etching of MoS_2_ flake films, which act as defect states within the MoS_2_ band gap, thereby degrading the device performance [15]. In addition, Vogl et al. reported the radiation tolerance of TMD-based devices using various irradiation sources, including gamma rays, protons, and electrons. The results indicated negligible variations in the current–voltage characteristics and photoluminescence intensity of mechanically exfoliated MoS_2_ devices that were subjected to gamma radiation [16]. Furthermore, Ozden et al. reported that the gamma radiation of few-layered MoS_2_ flakes created sulfur vacancies in the MoS_2_ structure, which were transformed into molybdenum oxide [17]. Moreover, Chen et al. performed gamma irradiation experiments on MoS_2_ transistors with solid polymer electrolyte gate dielectrics. After being subjected to gamma irradiation, the devices performed well; however, transfer curve changes and threshold voltage shifts were observed owing to the radiation-induced trapped electrons near the interface of the solid polymer electrolyte and MoS_2_ [18].

Although MoS_2_ materials offer promising prospects for downscaling integrated devices and circuit applications, the entire fabrication process is not fully compatible with the standard complementary metal–oxide–semiconductor (CMOS) process. Therefore, MoS_2_ synthesis on a high-quality large-area wafer scale is required. Chemical vapor deposition (CVD) has been identified as an effective method for producing high-quality TMD thin films on a wafer scale [19,20,21,22]. Most CVD synthesis methods involve providing transition metal sources, either in gas form or as predeposited metal or metal oxide precursor thin films on growth substrates [23,24,25,26]. The predeposition growth technique is a commonly used two-step process, in which transition metals (Mo and W) or transition metal oxides (MoO_3_ and WO_3_) are first predeposited on growth substrates and then sulfurized using a high-temperature thermal annealing process [27]. Although this simple two-step growth technique can provide large-area uniformity and layer-controllable growth, it is essential to transfer TMD thin films to target substrates to produce integrated electronic devices owing to the high-temperature CVD process [28]. In addition, the overall device performance is determined by certain crucial factors, including the contact resistance, dielectric scaling, and conformal gate controllability when designing device architectures [29,30]. Typically, MoS_2_ field-effect transistors (FETs) based on the conventional back-gate structure can achieve a nonuniform distribution for the gate electric field owing to the abnormal gate shape that occurs during the conventional lift-off process, which degrades the intrinsic electrical performance of 2D FETs. The implementation of a buried back-gate structure for uniform field induction improves the performance of the 2D MoS_2_-based FETs [31]. Furthermore, this gate geometry enables individual control of each device, which is essential for practical applications in CMOS-like logic devices. Therefore, understanding and analyzing the radiation responses of MoS_2_ devices are critical steps in the implementation of radiation-related electronic devices for practical applications. In contrast to MoS_2_ FETs with conventional top- or back-gate structures, the effects of gamma irradiation on the electrical performance of MoS_2_ FETs with a buried local back-gate structure have not been investigated (to the best of our knowledge).

In this study, we synthesized MoS_2_ film on the 2-inch wafer scale using two-step growth and fabricated MoS_2_ FETs with a buried local back-gate structure. The effects of gamma irradiation on the MoS_2_ FETs with a buried local back-gate structure were investigated. The electrical properties and gamma ray-induced changes in the MoS_2_ FETs before and after gamma irradiation were assessed. Furthermore, the possible interaction mechanism behind the electrical changes in the MoS_2_ and Al_2_O_3_ interface due to gamma irradiation was analyzed in detail.

## 2. Materials and Methods

### 2.1. Device Fabrication

A photoresist (PR, AZ 5214) was coated onto a SiO_2_ (100 nm)/Si substrate using a spin coater. The coated substrate was baked at 110 °C on a hot plate for 50 s before being exposed to ultraviolet light (MDS-400s) for gate patterning. Subsequently, PR was removed using a developer (AZ 300 MIF) for approximately 40 s. A dehydration bake was then carried out on a hot plate at 90 °C for 5 min to remove moisture from the surface. The SiO_2_ layer of the gate-patterned area was etched via reactive-ion etching (RIE) with CF_4_ gas (30 sccm, 100 W, and 2 min). The depth of the etched SiO_2_ layer was approximately 50 nm. Subsequently, a Ti/Au (5/50 nm) layer was deposited using an electron beam evaporator. An Al_2_O_3_ layer with a thickness of 30 nm was deposited as the gate dielectric layer via plasma-enhanced atomic layer deposition. A 2D MoS_2_ thin film was coated with polymethyl methacrylate (PMMA, KAYAKU A5) to transfer the synthesized MoS_2_ films onto the Al_2_O_3_ dielectric. The coated MoS_2_ film was etched in a 5% hydrogen fluoride solution for approximately 30 s before being rinsed thrice with deionized water for 5 min each time. The PMMA/MoS_2_ film was then carefully transferred onto a gate dielectric layer. The wet-transferred MoS_2_ film was baked at 110 °C for 12 h on a hot plate. Next, the substrate with the MoS_2_ film was soaked in acetone and isopropyl alcohol, and then annealed at 200 °C in a high-vacuum environment of 10^−6^ Torr for 1 h to remove the PMMA. Au masking was performed for channel patterning to minimize the effect of PR residue on the MoS_2_ film. After depositing Au with a thickness of 20 nm, a channel was formed using photolithography. The region, excluding the channel, was selectively etched using a Au etchant. The active channels of the MoS_2_ film were formed via RIE, and the residual PR was removed using acetone, which was followed by annealing under the same conditions as described previously. Finally, Au with a thickness of 50 nm was deposited using an electron-beam evaporator for the source/drain process using the same pattern formation process. Finally, an Al_2_O_3_ layer with a thickness of 30 nm was deposited as a passivation layer via atomic layer deposition.

### 2.2. Characterization of MoS_2_ Film

Focused-ion beam transmission electron microscopy (FIB-TEM) was used to confirm the layered MoS_2_ film structure on the SiO_2_ (100 nm)/Si substrate. In addition, energy-dispersive X-ray spectroscopy (EDS) mapping was used to determine the elemental composition (i.e., Mo, S, Si, and O) of the samples. The synthesized MoS_2_ film was examined via Raman spectroscopy using a NANOBASE XperRAM-CS Raman spectrometer with 532 nm laser excitation. Finally, the components and chemical composition of the MoS_2_ film were comprehensively examined via X-ray photoelectron spectroscopy (XPS) using NEXSA equipment.

## 3. Results and Discussion

### 3.1. Synthesis of MoS_2_ on SiO_2_ Substrate

**Figure 1:**
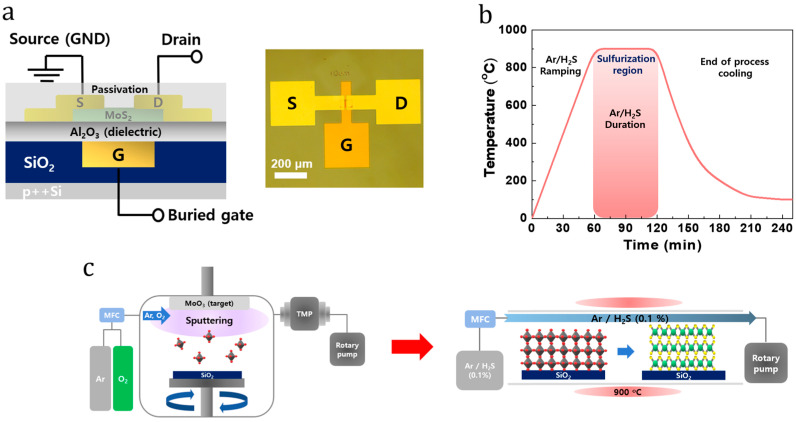
(**a**) Illustration and optical image of the fabricated MoS_2_ FET with a buried local back-gate structure. (**b**) Plot of temperature as a function of time for the thermal CVD sulfurization process. (**c**) Schematic of the two-step fabrication of the 2D MoS_2_ films.

Figure 1a shows an illustration and an optical image of the 2D MoS_2_ buried local back-gate device. Figure 1b shows the temperature profile for the sulfurization of MoS_2_ via thermal CVD. The working pressure was maintained at 800 Torr using Ar/H_2_S (0.1%) gas at a flow rate of 200 sccm. The process of sulfurization involved gradually increasing the temperature from room temperature to 900 °C over 1 h and then maintaining it at 900 °C for 1 h. After completing this process, the temperature in the tube naturally decreased to room temperature. The two-step synthesis of the MoS_2_ film is shown in Figure 1c. First, the SiO_2_ substrate was radio-frequency-sputtered with approximately 3 nm of MoO_3_, and then it was subjected to thermal CVD sulfurization. Thus, the MoS_2_ films were successfully synthesized on SiO_2_ wafers.

### 3.2. Material Characteristics of the MoS_2_ Thin Films on the SiO_2_ Wafers

**Figure 2:**
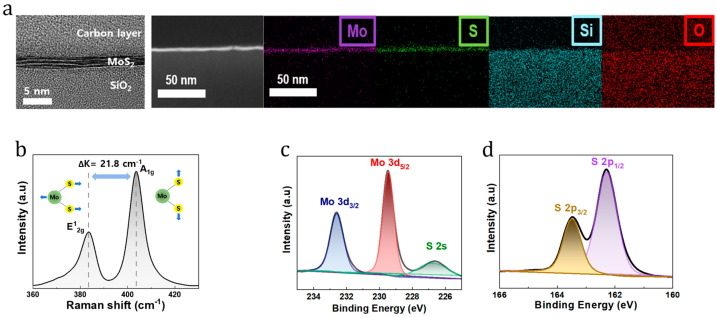
(**a**) FIB-TEM image of the 2D MoS_2_ film on the SiO_2_ substrate and EDS map of the 2D MoS_2_ multilayers. (**b**) Raman spectrum of the 2D MoS_2_ film on the SiO_2_ wafer. (**c**,**d**) XPS spectra of the MoS_2_ thin film.

FIB-TEM was used to confirm the vertically layered structure of the MoS_2_ film on the SiO_2_ substrate. The elemental composition of the film was analyzed via EDS mapping, which revealed the presence of Mo, S, Si, and O. As shown in Figure 2a, the synthesized MoS_2_ film was multilayered, atomically flat, and smooth. The crystal structure of MoS_2_ consists of Mo and S, whereas the SiO_2_ substrate is composed of Si and O. As shown in Figure 2b, the Raman spectrum of the MoS_2_ film on the SiO_2_ substrate revealed two large peaks at 381.8 and 403.6 cm^−1^, corresponding to the in-plane vibration of Mo and S atoms (E^1^_2g_) and out-of-plane (A_1g_) vibration of S atoms, respectively. The difference between the two MoS_2_ peaks was 21.8 cm^−1^, indicating that it was composed of four layers [32]. The MoS_2_ film components were further analyzed using XPS, as shown in Figure 2c,d. The XPS spectra of the Mo 3d and S 2s of the MoS_2_ film showed peaks at binding energies of 232.8 and 229.9 eV, corresponding to Mo 3d_3/2_ and Mo 3d_5/2_, respectively, whereas the peak at 227.1 eV represented S 2s. Two prominent peaks were observed in the XPS spectrum at 163.9 and 162.7 eV, corresponding to S 2p_3/2_ and S 2p_1/2_, respectively [33]. Additionally, the Mo:S composition ratio of MoS_2_ was calculated to be approximately 1:2.3, which is similar to the MoS_2_ stoichiometric ratio.

### 3.3. Gamma Irradiation Test Results for MoS_2_ Buried Local Back-Gate Transistors

**Figure 3:**
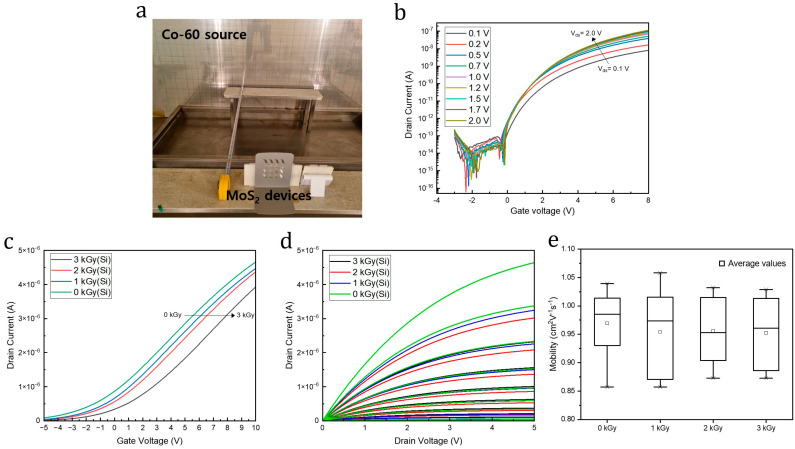
(**a**) Gamma irradiation test setup for MoS_2_ buried local back-gate transistors. Co-60 was used as the gamma source. (**b**) I_d_–V_g_ characteristics for the non-irradiated MoS_2_ buried local back-gate transistors. (**c**) I_d_–V_g_ characteristics, (**d**) I_d_–V_d_ characteristics, and (**e**) field-effect mobility of the MoS_2_ buried local back-gate transistors for various gamma irradiation doses.

Figure 3a shows the experimental setup for the gamma irradiation of the MoS_2_ samples. Irradiation experiments were performed in the irradiator facility at the Korea Atomic Energy Research Institute (KAERI). The Co-60 source, which comprises pencil-type modules, was kept in a pool of water when not in use and lifted out of the pool when needed for irradiation. The gamma rays from the Co-60 source had average energies of 1.17 and 1.33 MeV. The MoS_2_ samples were exposed to gamma radiation from the Co-60 source at a dose of approximately 1 kGy(Si)/h for various durations. Hereafter, the unit of kGy(Si) is denoted as kGy. All MoS_2_ samples were placed at a distance of 120 cm away from the Co-60 source according to the measured dose rates.

Current (I)–voltage (V) measurements were performed before and after radiation exposure to investigate the transient effects of gamma irradiation on the electrical properties of the MoS_2_ transistors. The transfer and output characteristics were measured at the total doses of 0 (non-irradiated), 1, 2, and 3 kGy. Figure 3b shows the transfer curves for the non-irradiated MoS_2_ transistors with gate voltages (V_g_) ranging from −3 to 8 V at a fixed drain voltage (V_d_) in the range of 0.1–2.0 V. The curves exhibit a typical n-type behavior, corresponding to the n-type electronic properties of the MoS_2_ semiconductor [8,34]. Moreover, the off-state current for V_g_ = −2 V was 10^−14^ to 10^−15^ A and the on-state current was approximately 10^−8^ A, indicating the efficient depletion of the device channel through the gate bias control. Thus, the on/off current ratio of the non-irradiated sample was 10^6^–10^7^, which is comparable to previously reported results and is sufficient for electronic applications such as switch devices [35].

Figure 3c shows the transfer curves for the MoS_2_ transistors before and after gamma irradiation for cumulative doses of 1, 2, and 3 kGy. The transfer characteristics were obtained by sweeping V_g_ from −5 to 10 V and setting V_d_ to 1 V. The current decreased as the gamma ray dose was increased. The current of the non-irradiated MoS_2_ transistors at 10 V decreased by approximately 15.7% after exposure to a gamma radiation dose of 3 kGy. The threshold voltages of −1.77, −1.27, −0.78, and −0.62 V were measured for the MoS_2_ transistors under exposures of 0, 1, 2, and 3 kGy, respectively. The positive threshold voltage shifts of 0.5, 0.99, and 1.15 V were observed for the MoS_2_ transistors under irradiations of 1, 2, and 3 kGy, respectively, compared to the non-irradiated device. Notably, 20 MoS_2_ FET devices were tested under gamma irradiation doses up to 3 kGy, with steps of 1 kGy, to verify data reliability. All MoS_2_ devices exhibited a similar trend of a positive shift in transfer characteristics. These positive shifts in the threshold voltages of the transfer curves are attributable to the comprehensive effects of hole accumulation in the Al_2_O_3_ dielectric interface near the MoS_2_ side and the formation of electron-trapping sites at the interface, which increased the electron tunneling at the MoS_2_ channel/dielectric interface. A more detailed explanation of the electrical changes caused by gamma irradiation is provided in Section 3.4.

Figure 3d shows the output curves for the MoS_2_ transistors before and after gamma irradiation at radiation doses of 1, 2, and 3 kGy, where V_d_ was swept from 0 to 5 V and V_g_ was varied from −5 to 3 V in steps of 1 V. As shown in the output characteristics, all MoS_2_ transistors exhibited drain-current saturation in the high-bias region and well-defined linear behavior in the low-bias region, indicating that the buried local back-gate bias completely controlled the channel carrier transport. Moreover, the drain current level decreased as the total gamma irradiation dose increased, indicating that gamma irradiation degraded both the channel quality and switching characteristics of the MoS_2_. As predicted, the degradation of the output characteristics followed a trend similar to that of the transfer characteristics. Although the degradation in the output current was observed after 1, 2, and 3 kGy of gamma irradiation, the channel conductance of all samples was efficiently modulated by the gate bias control, indicating that the switching characteristics between on and off states allowed for normal FET operations even after gamma irradiation.

To further investigate the effects of gamma rays on MoS_2_ channels, their field-effect mobilities ($\mu_{FE}$) were calculated using the following equation:

$$\mu_{FE}=\left(\frac{dI_{d}}{dV_{g}}\right)\left(\frac{L}{{WC_{ox}V}_{d}}\right)$$

where *L* represents channel length (20 μm), *W* represents channel width (50 μm), and *C_ox_* is the dielectric capacitance per unit area between the channel and the gate. As shown in Figure 3e, the average mobilities of 0.97, 0.95, 0.96, and 0.95 cm^2^V^−1^s^−1^ were observed for MoS_2_ transistors under gamma irradiation of 0, 1, 2, and 3 kGy, respectively. The result showed no significant differences between MoS_2_ devices before and after irradiation, indicating that the MoS_2_ layers could withstand gamma irradiation doses up to 3 kGy. Previous studies have reported that MoS_2_ layers can endure higher gamma radiation up to 10–130 kGy without any noticeable degradation in their properties [16,36,37].

### 3.4. Mechanism of Electrical Characteristics Changing According to Gamma Irradiation Dose

**Figure 4:**
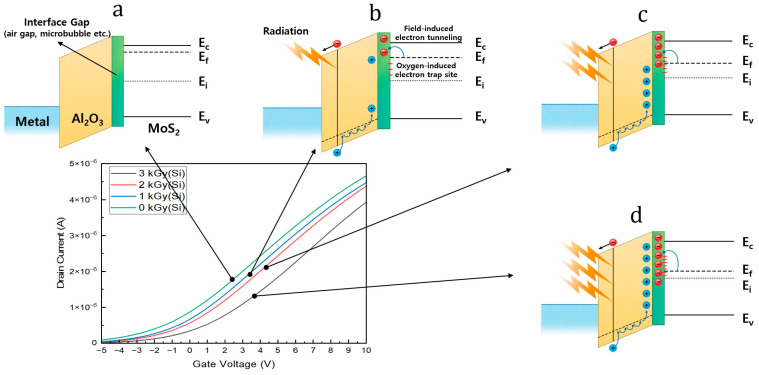
I_d_–V_g_ characteristics and band statistics of MoS_2_ buried local back-gate transistors under different gamma irradiation doses: (**a**) before irradiation, (**b**) under 1 kGy irradiation, (**c**) under 2 kGy irradiation, and (**d**) under 3 kGy irradiation.

Figure 4 shows the positive shift in the transfer curve as the gamma irradiation dose was increased from 0 to 3 kGy and the change in the band alignment of gate Al_2_O_3_–MoS_2_ under each condition. Similar to previously reported results [38], the transfer curve of the gamma-irradiated MoS_2_ transistor exhibited a positive shift. The mechanism for this phenomenon was analyzed. When a typical bulk metal–oxide–semiconductor (MOS) transistor is exposed to gamma rays, electrons and holes are generated in the oxide. Electrons escape the oxide at a high speed but holes cannot escape at a slow speed and are trapped at the interface, thereby degrading the transistor performance. Consequently, the off-current increases and shifts the transfer curve negatively owing to the positive field of the hole. When gamma rays are irradiated toward the MoS_2_ transistor, a positive shift, which is the opposite to that observed for the bulk semiconductor, occurs. This phenomenon has been reported as a carrier trapping/detrapping mechanism at the interface between 2D nanodevices and dielectrics [38,39,40,41,42], which can be explained in conjunction with the band diagram and Fermi-level shift. In an n-type MOS FET, the positive shift in the transfer curve signifies that more positive fields are needed to turn the transistor into the ON state. This result can be summarized in three situations: (1) the accumulation of a negative field source that interferes with the positive field in the gate oxide, (2) the intrinsic change in the Fermi level of the MoS_2_ channel owing to external factors, and (3) the trapping of electrons flowing in the channel at the interface. In the MoS_2_ transistor, all three of these mechanisms occur, and the primary causes are the air gap between the dielectric and MoS_2_ layers in the 2D nanodevice and the electron trap source that penetrates the grain boundary of the dielectric. When a gamma ray is incident on a MoS_2_ transistor, a hole is trapped in the surrounding dielectric interface, and electron tunneling occurs from the MoS_2_ channel to the surrounding air gap owing to the positive field of the hole. Consequently, the Fermi level of the MoS_2_ moves intrinsically [43]. Moreover, as the intensity of the gamma irradiation increases, the number of tunneled electrons increases. Therefore, as the magnitude of the gamma ray irradiation increases, the MoS_2_ channel changes intrinsically and the transfer curves undergo a positive shift. Additionally, gas molecules can penetrate the grain boundaries of the upper passivation layer [44], and a redox reaction occurs, causing MoS_2_ electrons to be used as a source. Furthermore, the by-product of the interfacial redox reaction is the OH^–^ group, which acts as a negative field source that prevents the positive field from accessing the gate. In addition, ozone created by the gamma rays in the atmosphere penetrates the grain boundaries and acts as an electron-trapping site [45]. The mechanisms described above produce a comprehensive effect, whereby a higher exposure to gamma rays results in more reactions and promotes a positive shift.

## 4. Conclusions

In this study, we successfully fabricated MoS_2_ FETs with a buried local back-gate structure and investigated the effects of gamma irradiation on the electrical performance of the devices. The transfer and output characteristics of the resulting devices were slightly degraded after gamma irradiation. Under a gamma irradiation dose of up to 3 kGy, the current level of the MoS_2_ transistors decreased by 15.7% compared to that of the non-irradiated devices. Moreover, the threshold voltage of the MoS_2_ transistors positively shifted by 0.50, 0.99, and 1.15 V after irradiation doses of 1, 2, and 3 kGy, respectively, compared to that of the non-irradiated devices. After analyzing the mechanisms involved, the positive shifts in the threshold voltages were attributed to the comprehensive effects of hole accumulation in the Al_2_O_3_ dielectric interface near the MoS_2_ side and the formation of electron trapping sites at the MoS_2_ channel/dielectric interface. Therefore, the results of this study provide a clearer understanding of the dominant factors that degrade the electrical properties of MoS_2_ transistors. The results also suggest directions for improving vulnerable regions and appropriate device configurations in high-radiation environments, such as those relevant to space, nuclear, and aerospace applications.

## Data Availability

Data will be made available on request.
